# Variable expression of cysteinyl leukotriene type I receptor splice variants in asthmatic females with different promoter haplotypes

**DOI:** 10.1186/1471-2172-10-63

**Published:** 2009-12-15

**Authors:** Milena Sokolowska, Karolina Wodz-Naskiewicz, Malgorzata Cieslak, Karolina Seta, Andrzej K Bednarek, Rafal Pawliczak

**Affiliations:** 1 Department of Immunopathology, Chair of Allergology, Immunology and Dermatology, Faculty of Medical Science and Postgraduate Training, Medical University of Lodz, Pomorska 251 str, 92-213 Lodz, Poland; 2 Department of Immunology, Rheumatology and Allergy, Chair of Clinical Immunology and Microbiology, Faculty of Medicine, Medical University of Lodz, Pomorska 251 str, 92-213 Lodz, Poland; 3 Department of Molecular Carcinogenesis, Chair of Molecular Medicine and Biotechnology, Faculty of Medical Science and Postgraduate Training, Medical University of Lodz, Mazowiecka 6/8 str, 92-215 Lodz, Poland

## Abstract

**Background:**

Cysteinyl leukotrienes are potent inflammatory mediators implicated in the pathogenesis of asthma. Human cysteinyl leukotriene receptor 1 (*CYSLTR1*) gene contains five exons that are variably spliced. Within its promoter few polymorphisms were described. To date, there has been no evidence about the expression of different splice variants of CysLT_1 _in asthma and their association with *CYSLTR1 *promoter polymorphisms.

The goal of our study was to investigate CysLT_1 _alternative transcripts expression in asthmatic patients with different *CYSLTR1 *promoter haplotypes.

The study groups consisted of 44 patients with asthma, diagnosed according to GINA 2008 criteria and 18 healthy subjects. Genomic DNA and total RNA was extracted from peripheral blood mononuclear cells. Real-time PCR was performed with specific primers for transcript I [GenBank:DQ131799] and II [GenBank:DQ131800]. Fragments of the *CYSLTR1 *promoter were amplified by PCR and sequenced directly to identify four single nucleotide polymorphisms: C/T [SNP:rs321029], A/C [SNP:rs2637204], A/G [SNP:rs2806489] and C/T [SNP:rs7066737].

**Results:**

The expression of CysLT_1 _transcript I and II in asthma did not differ from its expression in healthy control group. However, in major alleles homozygotic CAAC/CAAC women with asthma we found significantly higher expression of transcript I as compared to heterozygous CAAC/TCGC women in that loci. CysLT_1 _transcript I expression tended to negative correlation with episodes of acute respiratory infection in our asthmatic population. Moreover, expression of CysLT_1 _transcript II in CAAC/CAAC homozygotic women with asthma was significantly lower than in CAAC/CAAC healthy control females.

**Conclusions:**

Genetic variants of *CYSLTR1 *promoter might be associated with gender specific expression of CysLT_1 _alternative transcripts in patients with asthma. CysLT_1 _splice variants expression might also correlate with the susceptibility to infection in asthmatic population.

## Background

Asthma is a complex disease influenced by genetic and environmental factors [1], with highly variable clinical spectrum, but with the consistent presence of airway inflammation. Among many important inflammatory mediators involved in its pathogenesis are cysteinyl leukotrienes. They are potent lipid mediators derived from the lipooxygenase pathway of arachidonic acid metabolism [2,3]. They are products of activated eosinophils, mast cells, basophils and macrophages [3,4]. Originally identified as potent mediators of bronchoconstriction, mucus secretion, and airway hyperresponsiveness [2,5], they are now also described as important factors of innate and adaptive immune responses [6], as well as in the effector phase of inflammation, tissue repair and fibrosis [7,8]. So far, three cysteinyl leukotrienes receptors have been identified: CysLT_1_[9,10], CysLT_2 _[11-13] and recently described GPR17 [14]. All of them are seven transmembrane-spanning receptors that couple to G proteins (GPCR) and activate various intracellular signaling pathways [15]. The distribution of these three types of CysLTs receptors is diverse and depends on the cells and tissues as well as on the pathophysiologic conditions. In human lungs CysLT_1 _protein was demonstrated in the smooth muscle cells at all levels of the respiratory tract, interstitial lung macrophages and in the epithelial cells as well as in infiltrating inflammatory cells such as monocytes/macrophages, mast cells, eosinophils, CD 34+ cells, neutrophils and B lymphocytes [9,10,16,17]. CysLT_1 _mRNA was observed in normal and asthmatic human lungs [10,16]. However, recent studies have shown a significant increase in CysLT_1 _positive cells in the bronchi of stable asthmatics in comparison to healthy, non smoking controls and further elevation of CysLT_1 _mRNA and protein in acute severe exacerbations of asthma [18]. Nevertheless, response to leukotriene antagonists (LTRA) in asthma is known to be variable and it still has not been determined which of the phenotypes benefits most from this type of treatment [19-21]. Moreover, there is still a discrepancy about the precise location of the *CYSLTR1 *promoter region and the exon/intron structure of this gene [22,23]. Therefore, there is a possibility that at least two alternative promoters exist and might initiate different transcripts of the receptor gene in various pathophysiological conditions [22]. Furthermore, functional and case-control studies of the *CYSLTR1 *gene contribution to asthma have shown inconsistent results [22,24,25]. We have also previously observed [26] that some clinical features of severe asthma were associated with the minor [TCGC] haplotype of *CYSLTR1 *promoter SNPs in severe asthmatic women. However, we have found no significant differences in gene reporter activity among all tested promoter SNPs constructs. Therefore in this study we hypothesized that *CYSLTR1 *gene promoter polymorphisms might influence on the expression of CysLT_1 _alternative transcripts, which might further influence on the course of the disease. To test this hypothesis, we analyze the expression of CysLT_1 _alternative transcripts in patients with asthma and in healthy control group with the relevance to the *CYSLTR1 *gene promoter polymorphisms. We demonstrate here that there is no difference in the expression of CysLT_1 _alternative transcripts between asthmatic and healthy population analyzed without focus on genetic background and sex stratification.

However, there is a difference in the CysLT_1 _alternative transcripts expression in women with asthma with different promoter haplotypes. Furthermore, we also demonstrate that the expression of certain splice variants correlates with the episodes of acute infections.

## Materials

### Study population

The study involved 44 patients with persistent asthma randomly selected from asthmatic group treated in the Outpatient Unit of the Department of Immunology, Rheumatology and Allergy in Lodz, Poland. The sex matched healthy individuals group consisted of 18 unrelated, non-atopic subjects with a negative family history of allergy and asthma. Asthma was defined by a specialist of respiratory diseases according to GINA 2008 criteria [27]. Atopy was defined as one or more positive reactions in the skin prick tests with the battery of inhaled allergens. The aspirin intolerant asthma phenotype was defined in patients having a proven history of asthma aggravation on exposure to aspirin/NSAIDs and a positive response in a lysine-aspirin bronchoprovocation test. Exacerbations were defined as acute worsening of asthma symptoms with decrease of FEV_1 _or PEF ≥ 20% diagnosed by the doctor and put in the case history as a word "exacerbation", with hospitalization or urgent visit for asthma or stated aggravation of asthma symptoms requiring at least doubling the dose of ICS or addition or periodic increase of oral GCS. Episodes of acute respiratory tract infection were defined as acute aggravation of patient's health with fever, yellow sputum and need for antibiotic usage with or without asthma exacerbation. Nocturnal dyspnoea was characterised and stated in the case history by the doctor according to the interview with the patient. Chronic rhinosinusitis was diagnosed on the basis of patients' symptoms and CT scans. Allergic rhinitis was defined according to ARIA 2008 criteria [28]. GERD was diagnosed according to American College of Gastroenterology 2005 recommendations [29]. The doses of ICS were calculated as the budesonide equivalent within the whole period of observation in the Outpatient Unit and divided by the number of observation days and presented as micrograms per day. Some patients received ICS by nebulization. The doses of systemic GCS were calculated as a prednisone equivalent. GCS, montelukast and zafirlukast were also divided by the number of whole days of observation and presented as milligrams per day. These drugs were prescribed to the patients for certain periods *e.g*. 2-3 weeks in regards of GCS in decreasing dosage in case of exacerbation or 3-4 months in regards of leukotriene receptor antagonists in case of worse asthma control and there were periods free of certain drugs. Therefore, median dose of certain drug per day includes periods without this drug usage. Thus, the dose of the drug could be lower than available and commonly used doses.

Clinical features of patients were analysed retrospectively over the whole period of observation using individually self-created database software, with the possibility of calculations of studied variables.

Characteristics of the studied groups are presented in Table 1. The study was approved by the Ethics Committee of the Medical University of Lodz and informed consent was obtained from every subject prior to the study.

**Table 1:** Characteristics of the studied groups

	Asthma	Controls
***n***	44	18
**Men/women [*n*]**	19/25	11/7
**Age [years]**	53 (21-77)	41(20-71)
**Period of observation [months]**	62 (9-124)	n.a.
**FEV_1 _[%]**	79 (37-113)	n.a.
**PEF [l/s]**	365 (223-610)	n.a.
**Exacerbations per year**	0.97 (0-3.31)	n.a.
**Episodes of acute infection per year**	0.49 (0-2.51)	n.a.
**Hospitalization for asthma per year**	0 (0-0.83)	n.a.
**Nocturnal dyspnoe per year**	0.36 (0-3.16)	n.a.
**ICS [μg per day]**	1258 (158-3289)	n.a.
**Oral GCS [mg per day]**	1.6 (0-10.6)	n.a.
**Oral GCS [days of use per year]**	76.4 (0-365)	n.a.
**Montelukast [mg per day]**	2.3 (0-10)	n.a.
**Zafirlukast [mg per day]**	0 (0-26.1)	n.a.
**Smokers [%]**	20.5	16.6
**Atopy [%]**	63	0.0
**GERD [%]**	2.6	0.0
**Chronic rhinosinusitis [%]**	20.5	0.0
**Aspirin intolerance [%]**	10.2	0.0
**Allergic rhinitis [%]**	59	0.0

Variables are shown as median and range or percentage. n.a. - non applicable.

### Genomic DNA Preparation and Single Nucleotide Polymorphisms (SNPs) Genotyping

Genomic DNA was extracted from peripheral blood mononuclear cells of studied subjects using Genomic Maxi AX (A&A, Gdansk, Poland). A 633 bp fragment containing [SNP:rs321029] C/T, [SNP:rs2637204] A/C was obtained with the following primers: 5'-AGCCTTTTCCTTTCTGGTTC-3'(forward) and 5'-TCACCATCACCACCACAATC-3'(reverse) (Tib Molbiol, Poznan, Poland). A 602 bp fragment containing the [SNP:rs2806489] A/G and [SNP:rs70667337] C/T was amplified with the following primers: 5'-GAGCAGGGCACATTTGCTAG-3'(forward) and 5'-GCAAGCCCAGTCATTCCAGA-3'(reverse) (Tib Molbiol). Primer design and optimization of annealing temperatures were performed with MacVector software (Oxford Molecular Group, Madison, WI). PCR condition were as follows: initial 5 min at 95°C followed by 35 cycles of 30 s at 94°C, 30 s at 58°C, 45 s at 72°C and 7 min at 72°C. The PCR reactions were performed in Biometra T3 thermocycler (Biometra, Göttingen, Germany). These PCR fragments were purified with Clean-Up (A&A) and labeled using fluorescently labeled dye terminators technique (Applied Biosystems, Foster City, CA) with specific nested primers. To analyze sequence including rs321029 and rs2637204, following primers were used: 5'-ACATCAAAGTGCTGCCCCAG-3' and for sequence including rs2806489 and rs70667337: 5'-GGAACCAGAAAGGAAAAGGC-3' (Eurogentec, Polgen, Lodz, Poland) and appropriate primers from PCR in case of samples from female subjects which were sequenced in both directions. Second purification step was performed using ExTerminator kit (A&A). All sequencing reactions were performed in ABI Prism 310 capillary sequencer (Applied Biosystems).

### Real-Time PCR analysis of alternative transcripts expression

Peripheral blood mononuclear cells were isolated from the blood using Histopaque 1077 (Sigma-Aldrich, Poland) density gradient centrifugation. Total RNA was isolated from peripheral blood mononuclear cells using the Total RNA Extraction Kit (A&A) according to manufacturer's protocol in the presence of RQ1 RNase-Free DNase (Promega, Madison, WI, USA). Total RNA (2 μg) was reverse-transcribed using the random hexamers and ImProm-II™ reverse transcription system (Promega) in a volume of 25 μl at 37°C for 90 min. All real-time PCR reactions were optimized to obtain the best amplification kinetics. Amplification reactions were performed in a final volume of 20 μl using qPCRTM Core Kit for SybrTM Green I w/o dUTP (EUROGENTEC, Seraing, Belgium), 250 nM primers and 25 ng cDNA template per reaction. Nuclease free water was used as a no-template control. The following primer pairs were used: for CysLT_1 _transcript I [GenBank:DQ131799]: forward 5'-AACGCAAAAGGACAGTAAACTGTG-3' and reverse 5'-ATCAATGCCTTTTACGGTGTAATATTAG-3'; for CysLT_1 _transcript II [GenBank:DQ131800]: forward 5'-TCGAATTTACTGAAGACTTGGAGCTTGCTTC-3' and reverse 5'-TCTCTACGAATGTCTGCTTTGTGCCTGC-3', for RPS17: 5'-ACCCCAATGTCAAGGAGATCAAGGTCCTG-3' and 5'-TCGGCAGCCAGCTCGTGAGTAATG-3', for histone H3A - H3F3A: 5'-AGGACTTTAAAAGATCTGCGCTTCCAGAG-3' and 5'-ACCAGATAGGCCTCACTTGCCTCCTGC-3'. Gene expression levels of CysLT_1 _transcript I and II and two reference transcripts: RPS17, and H3F3A were tested using the Rotor-Gene 3000 Real-Time DNA analysis system (Corbett Research, Australia). Cycling conditions: 1 cycle at 95°C for 10 min, 40 cycles at 95°C for 30 s, 56°C for 15 s (CysLTR1 transcript I) or 65°C for 15 s (CysLT_1 _transcript II) or 64°C for 15 s (RPS17) or 65°C for 15 s (H3F3A), and 72°C for 30 s. The relative expression was based on the expression ratio of the target gene *versus *reference genes (RPS17 and H3F3A). To calculate the relative expression ratios of single samples a mathematical model was used which included an efficiency correction for the real-time PCR efficiency of the individual transcripts [30]. Relative expression was calculated using REST (Corbett Research, Australia) software with the two reference genes mentioned above. Each experiment was conducted in triplicate.

### Statistical analysis

The data from the study were analyzed using Statistica (v. 6.0; StatSoft, Tulsa, OK). The distribution of all examined variables was checked for normality. Because of the deviation from normal distribution, in the analysis of alternative transcripts expression U-Mann-Whitney test was performed to analyze differences between variables. Data are expressed as mean relative expression ± SEM. Relationship between alternative transcripts expression and studied asthmatic features were evaluated utilizing Spearman's rank correlation test. Haplotype frequencies and linkage disequilibria were estimated using the HaploView 4.0 program [31]. Haplotypes of each individual were inferred by using the algorithm developed by Stephens *et al*. (PHASE version 2.1) [32]. The *post-hoc *analysis of the quantitative variables with the Bonferroni correction was performed to account for multiple testing.

## Results

### Study population

We analyzed the expression of CysLT_1 _alternative transcript I and II in 44 patients with asthma and 18 healthy controls. Characteristics of study populations are shown in Table 1. Haplotype distributions in both analyzed populations are shown in Table 2. CAAC and TCGC were dominant haplotypes in patients with asthma and in healthy controls. The relative expression of CysLT_1 _transcript I in peripheral blood mononuclear cells was similar in asthma (22.87 ± 5.93) and in healthy control group (26.23 ± 12.98) (p > 0.05). Moreover, relative expression of CysLT_1 _transcript II was lower than transcript I and also similar in asthma (5.05 ± 0.84) and in controls (8.54 ± 2.41) (p > 0.05). Furthermore, the expression of these two splice variants did not differ either between female patients with asthma and female controls or male patients *vs *male controls.

**Table 2:** Haplotypes frequency in the asthmatic population (n = 44) and healthy subjects (n = 18)

Alleles	C/T (rs321029)	A/C (rs2637204)	A/G (rs2806489)	C/T (rs70667337)	%
**Asthmatic population (n = 44)**					

Haplotype					
ht1	C	A	A	C	75.4
ht2	T	C	G	C	20.3
ht3	C	A	A	T	1.4
ht4	C	A	G	C	1.5
ht5	T	C	A	C	1.4

**Healthy subjects (n = 18)**					

Haplotype					
ht1	C	A	A	C	84
ht2	T	C	G	C	12
ht3	C	A	A	T	4
ht4	C	A	G	C	0
ht5	T	C	A	C	0

### Alternative Transcripts-Haplotype Association Study

**Figure 1:**
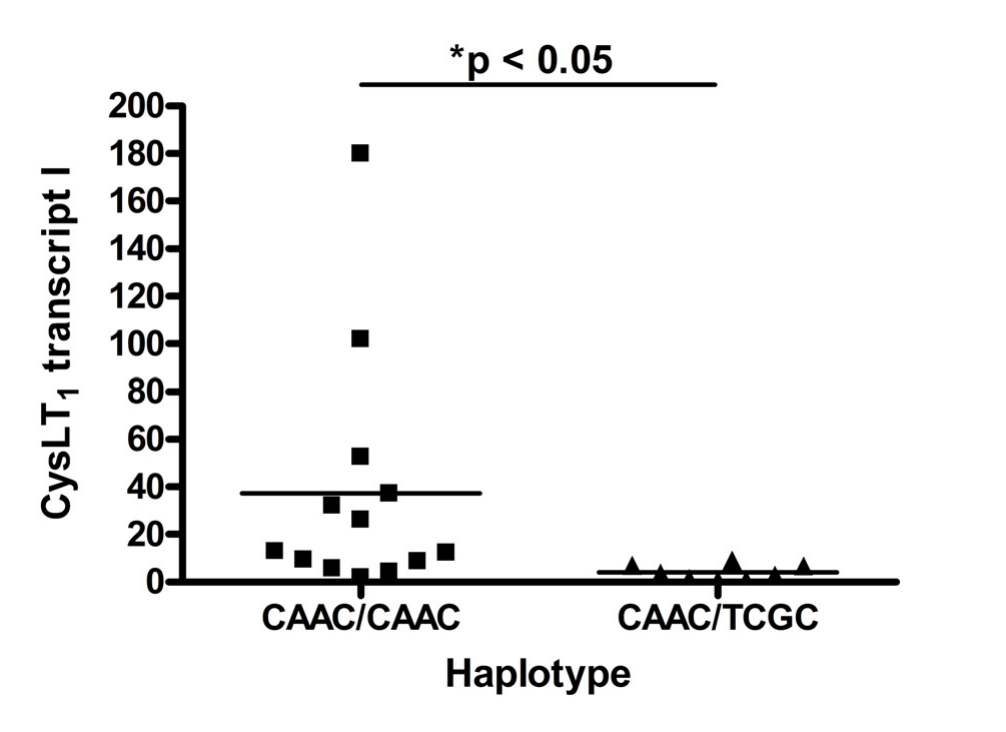
**CysLT_1 _transcript I relative expression in women with asthma with two main *CYSLTR1 *haplotypes**. Data are expressed as dots. Horizontal line represents mean. Asterisk indicates p < 0.05.

**Figure 2:**
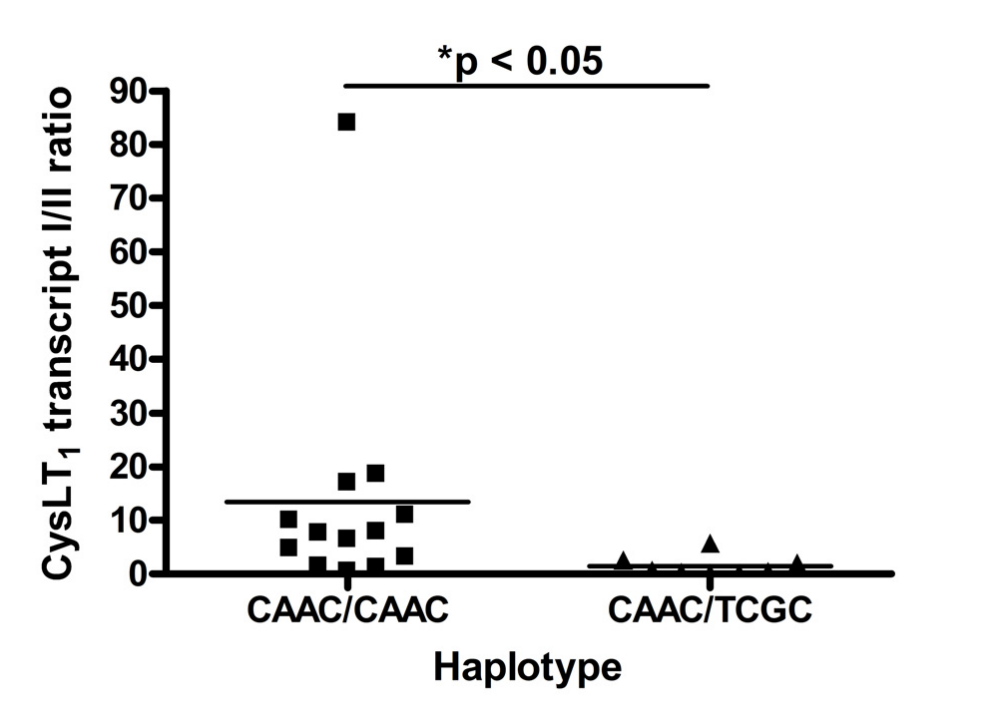
**CysLT_1 _transcript I/II relative expression ratio in women with asthma with two main *CYSLTR1 *haplotypes**. Data are expressed as dots. Horizontal line represents mean. Asterisk indicates p < 0.05.

We hypothesized that the different expression of CysLT_1 _alternative transcripts might depend on the polymorphisms in the *CYSLTR1 *gene promoter region. Thus, we performed an analysis concerning association of the CysLT_1 _splice variants expression with the haplotypes in each locus within the whole study population. We analyzed the differences between the two main haplotypes CAAC and TCGC as they accounted for nearly 90% of the studied populations, which was shown in Table 2. The other haplotypes were too rare to perform appropriate analysis. In women with asthma who are homozygotes in major alleles in each analyzed locus CAAC/CAAC we found significantly higher expression of transcript I 37.22 ± 14.11 (p_cor _= 0.0169) and transcript I/II 13.38 ± 6.10 ratio (p_cor _= 0.009) as compared to heterozygous CAAC/TCGC women in that loci, 4.08 ± 1.06 and 1.49 ± 0.65, respectively (Figure 1, 2). We did not observe any differences in alternative transcripts expression in asthmatic men with different promoter genotypes or in both sexes of healthy patients.

### CysLTR1 alternative transcripts correlations

**Figure 3:**
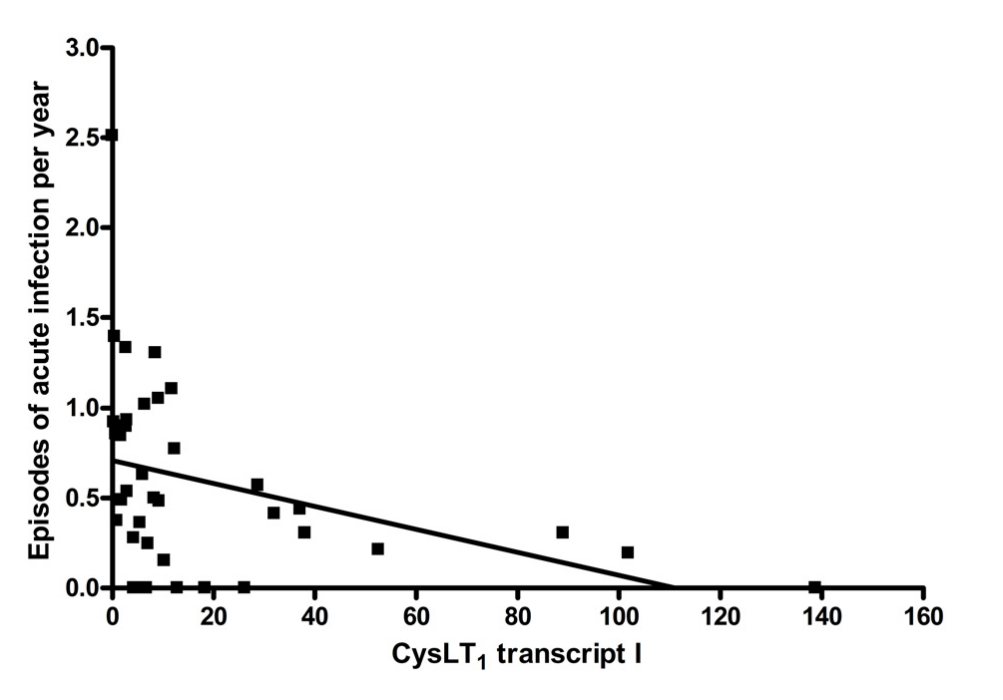
**Correlation of CysLT_1 _transcript I relative expression with the episodes of acute infection in the whole asthmatic population**. Spearman's rank correlation coefficient was R = - 0.52, p_cor _= 0.0009.

We analyzed here correlations of CysLT_1 _alternative transcripts expression with different features of asthma such as FEV_1_, PEF, exacerbations, episodes of acute infection, hospitalization for asthma, nocturnal dyspnoe and intake of ICS, systemic GCS and LTRA in the whole asthmatic population. We observed weak but statistically significant negative correlation of CysLT_1 _transcript I expression with episodes of acute infection (R = - 0,52, p_cor _= 0.0009) (Figure 3). Relative expression of CysLT_1 _transcript II did not correlate with any of studied asthmatic features.

### Alternative Transcripts-Haplotype-Phenotype Association Study

**Figure 4:**
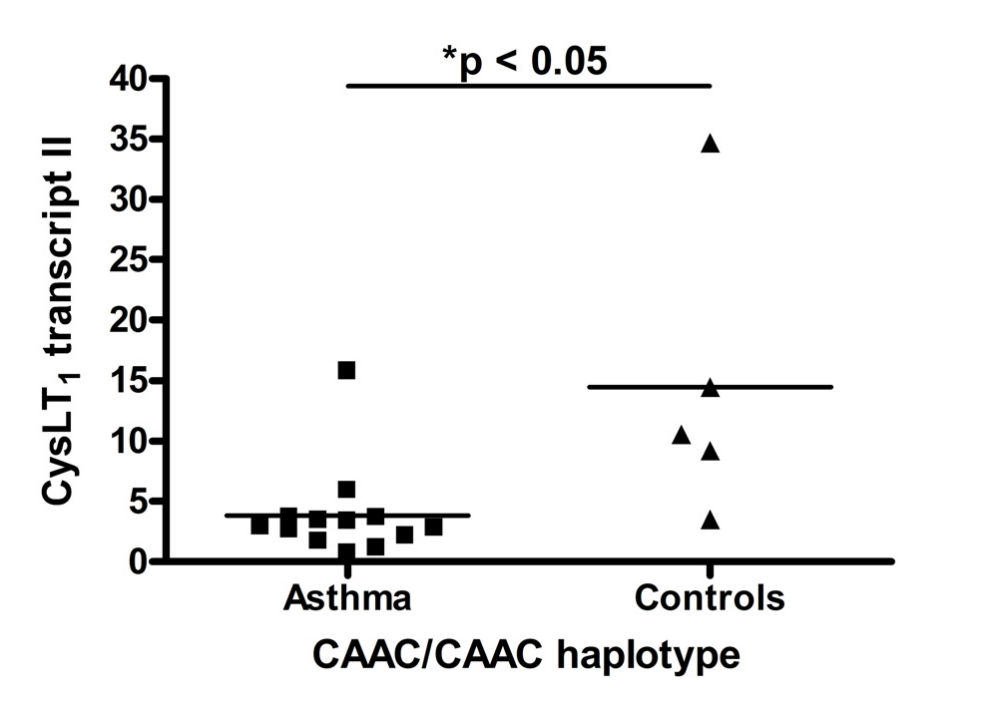
**CysLTR_1 _transcript II expression in CAAC/CAAC asthmatic and control females**. Data are expressed as dots. Horizontal line represents mean. Asterisk indicates p < 0.05.

We analyzed here if there are differences in CysLT_1 _splice variants I and II expression in carriers of the same haplotypes between asthmatic patients and healthy controls with subsequent sex stratification. Again, we took into consideration two main haplotypes CAAC and TCGC. We observed that relative expression of CysLT_1 _transcript II in CAAC/CAAC homozygotic women with asthma (3.86 ± 1.06) was significantly lower than in CAAC/CAAC healthy control females (14.48 ± 6.90) (p_cor _= 0.019) (Figure 4). There were no differences in case of CysLT_1 _transcript I expression in either of analyzed configurations.

## Discussion

Here we showed that there were no differences in CysLT_1 _alternative transcripts expression in the blood mononuclear cells between asthmatic patients and controls, when they were evaluated as genetically homogeneous populations and without sex stratification. Figueroa *et al *found that 20% of peripheral blood leukocytes showed the presence of CysLT_1_. These are cells of particular relevance to asthma and atopy, such as monocytes/macrophages, eosinophils, pregranulocytic CD34+ cells, neutrophils, and subsets of B lymphocytes [33] and probably also T lymphocytes [17]. According to our best knowledge there has been no data published comparing CysLT_1 _mRNA or CysLT_1 _alternative transcripts expression in peripheral blood mononuclear cells between asthmatic and healthy subjects. There might be a few reasons of lack of differences in the overall CysLT_1 _mRNA expression at least in the blood mononuclear cells. First of all, our patients were in the stable period of the disease, without a trace of exacerbation in the moment of blood collection. Therefore, inflammation might be present only locally in the bronchi, not in the peripheral blood, mainly because of the persistent influx of inflammatory cells from the blood to the bronchi. Zhu and colleagues found that there is an increase of distinct inflammatory cells expressing the CysLT_1 _receptor in the bronchial mucosa of mild asthmatic patients as compared to healthy subjects and further increase when there is a severe exacerbation of asthma [18]. However they did not analyze peripheral blood [18].

Nevertheless, it might also confirm our main hypothesis of indirect promoter SNPs influence on *CYSLTR1 *gene transcription and/or alternative splicing in a sex related manner. This might further correlate with the level of CysLT_1 _protein expression and finally with the disease phenotype. Several lines of evidence support this hypothesis. First of all, we found that heterozygotic females CAAC/TCGC with asthma had significantly lower expression of CysLT_1 _transcript I as compared to major female asthmatic homozygotes CAAC/CAAC (Figure 1, 2). This correlation was not observed in healthy women neither in asthmatic and healthy men. In our previous study on the association of these *CYSLTR1 *promoter SNPs with severe asthma [26] in the population of 93 severe asthmatics, 110 non-severe asthmatics and 100 healthy controls we showed that heterozygotic CAAC/TCGC females with severe asthma had significantly more episodes of infection per year, more exacerbations per year, more hospitalizations for asthma per year, and they used more ICS than homozygotic female carriers of the main haplotype CAAC/CAAC. However, we found no evidence on the influence of these promoter SNPs on the gene expression by means of reporter gene studies. Therefore, our current findings might at least partially link this association with the functional effects. Moreover, we showed here that CysLT_1 _alternative transcripts expression might slightly affect factors associated with the course of asthma. We have observed the weak tendency that the more episodes of acute respiratory infection per year in patients with asthma (Figure 3) the lower expression of CysLT_1 _transcript I. However, the feeble distribution of data limits any potential conclusions. Nevertheless, it could indicate on the probability of impaired innate and afferent mechanisms of acquired immune response in patients with asthma, probably through altered CysLT_1 _receptor expression [6]. CysLTs and their receptors were proven to be critical determinants of dendritic cells homing to regional lymph nodes and the repertoire of cytokines required to induce T cell responses [34,35]. It was shown that maturation of dendritic cells with LPS, a major endotoxin of Gram-negative bacteria and a classic Toll-like receptor 4 agonist, reduced CysLT_1 _receptor expression by 50% [36]. Moreover, generation of leukotrienes from white blood cells was enhanced during sepsis, and further increased after stimulation but only in patients who survived this massive infection [37]. This data might indicate the importance of leukotrienes and their receptors not only in the development of chronic inflammation in asthma but also in episodes of acute respiratory infections, which are one of the most important determinants of asthma exacerbations.

Episodes of acute respiratory infection in our study population by means of symptoms and reaction to antibiotic treatment fulfilled the definition of bacterial infection. The limitation of this definition is lack of exact evidence on bacterial or viral involvement. On the other hand, it has been proven that viral infections, like respiratory syncytial virus or parainfluenza virus, increase the level of cysteinyl leukotrienes [38-40] and that antileukotriene drugs alleviate symptoms of viral upper respiratory tract infections [41,42]. The last but not least argument for our initial hypothesis is that we have noticed that in CAAC/CAAC women with asthma CysLT_1 _transcript II is differentially expressed as compared to CAAC/CAAC healthy control women (Figure 4). Together with our previous findings that promoter SNPs did not influence its basal activity, these data might suggest that there are other factors *e. g*. splicing factors, sex hormones or the effects of some inflammatory cytokines that influence the *CYSLTR1 *gene transcription and/or splicing. It stays in agreement with primarily contradictory findings of our and other groups. Minor alleles of *CYSLTR1 *promoter SNPs increased [24,25] or decreased [22] or demonstrated no influence on the *CYSLTR1 *basic promoter activity [26]. Nowadays, there is an emerging consensus suggesting that splicing is in most cases initiated cotranscriptionally and that introns are removed while the nascent transcript is still tethered to the DNA [43]. Some transcriptional coregulators, brought to the promoters by transcription factors, either harbor splicing activity *e.g*. hnRNP [44] or interact with spliceosome components [45]. Inversely, some proteins like SKIP described initially as RNA-binding splicing factors were later shown to be present at promoters and/or to function as transcriptional regulators [46]. Four different mRNA CysLT_1 _transcripts were observed in human cells, with the dominant expression of transcript I in both unstimulated and IL-4 stimulated cells and far less abundant expression of transcript II. Transcript III and IV were found only in THP1 cell lines. However, the role of alternative transcripts of CysLT_1 _receptor has not been elucidated [23]. They all form the same protein structure as the coding region and ORF is included in the fifth exon, present in each transcript. The putative *CYSLTR1 *promoter was found to contain two STAT6 consensus response elements, GATA and AP1 binding sites [23]. Four analyzed here *CYSLTR1 *promoter SNPs do not change any known transcription factor binding sites, but they might influence on the splicing binding factors. Altered ratio of main transcripts I and II might further influence on the expression status of structurally unchanged receptor protein, not only in blood leukocytes but also in inflammatory infiltration of the bronchi. This subsequently could correlate with the phenotype. Elevated expression of CysLT_1 _plays a role in asthma and its exacerbation as shown Zhu *et al *[18]. Alternative splicing of pre mRNA represents a widespread mechanism for increasing variability of eukaryotic gene expression and has been associated with human pathologies, such as cancer, Alzheimer's, amyotrophic lateral sclerosis, ataxia teleangiectasia, cystic fibrosis, and other [47,48]. For instance, non functional alpha subunit of the epithelial sodium channel alternatively spliced forms have been proposed to serve as negative regulatory components for its activity in humans [49].

Finally, our previous and current data indicated the gender differences in cysteinyl leukotrienes and their receptors metabolic pathways. Female sex and a certain haplotype might be a risk factor of the disease. Lack of the TCGC haplotype effect on the CysLT_1 _alternative transcripts expression in both asthmatic and healthy men might suggest an additional effect of sex hormones or some behavioral/environmental differences. Recently, it has been shown in the large European multi-centered cohort of patients that severe asthma occurs four times more often in women that in men [50]. Moreover, in accordance of our observations very recently Pergola *et al *showed higher expression of 5-LO, 15-H(P)ETE and CysLTs in stimulated whole blood and neutrophils in females comparing to males accompanied with the differences in 5-LO cellular localization and trafficking [51]. These effects were further abolished by male hormones testosterone and 5-α-dihydrotestosterone [51]. Moreover, it has been shown that estradiol modulates the pulmonary influx of inflammatory cells, increases generation of IL-4 and mediates mast cell degranulation [52] while progesterone upregulates IL-4 production [53]. All of these effects might increase the number of cells with CysLT_1 _receptor in the bronchial mucosa of female subjects. Expression of *CYSLTR1 *gene may be also affected by exposure to different proinflammatory cytokines. It has been shown that CysLT_1 _may be upregulated in different cells by proasthmatic cytokines such as IL-4, IL-5 and IL-13 [54] and the influence of analyzed haplotypes on gene expression and alternative splicing may only be present under specific inflammatory conditions.

In summary, still little is known about factors involved in the processes of *CYSLTR1 *transcription, alternative splicing, and their influence on the final CysLT_1 _receptor protein expression and further correlation with the asthma phenotype. However, our data suggest that indeed *CYSLTR1 *gene might be implicated in asthma pathogenesis at least by differential expression of CysLT_1 _alternative transcripts in various haplotypes and in sex-related manner, but the exact mechanisms remain to be clarified.

## Conclusion

Genetic variants of *CYSLTR1 *promoter might be associated with gender specific expression of CysLT_1 _transcripts in patients with asthma. CysLT_1 _splice variants expression might also correlate with the susceptibility to infection in asthmatic population.

## Authors' contributions

MS participated in the study design, performed experiments, interpreted the data and wrote the manuscript. KWN and KS performed experiments and drafted the manuscript. AKB and RP contributed funding and materials to the study, helped to design the experiments, analyzed the data and critically revised the manuscript adding important issues. MC enrolled patients to the study, participated in its design and coordination and helped to draft the manuscript. All authors read and approved the final manuscript.
